# Editorial: Recent Advances in the Controversial Human Pathogens *Pneumocystis*, Microsporidia and *Blastocystis*

**DOI:** 10.3389/fmicb.2021.701879

**Published:** 2021-08-02

**Authors:** Olga Matos, Lihua Xiao

**Affiliations:** ^1^Medical Parasitology Unit, Group of Opportunistic Protozoa/HIV and Other Protozoa, Global Health and Tropical Medicine, Instituto de Higiene e Medicina Tropical, Universidade NOVA de Lisboa, Lisboa, Portugal; ^2^Faculdade de Medicina, Instituto de Saúde Ambiental, Universidade de Lisboa, Lisboa, Portugal; ^3^Center for Emerging and Zoonotic Diseases, College of Veterinary Medicine, South China Agricultural University, Guangzhou, China; ^4^Guangdong Laboratory for Lingnan Modern Agriculture, Guangzhou, China

**Keywords:** opportunistic protists, *Pneumocystis*, microsporidia, *Blastocystis*, epidemiology, diagnosis, therapy

*Pneumocystis* spp. are ubiquitous atypical fungi that develop extracellularly in the alveolar cavities of mammalian lungs with a not-completely defined lifecycle (Cushion, 2010). Similarly, microsporidia are a diverse group (>1,400 species) of obligate intracellular fungi, with a broad range of vertebrate and invertebrate hosts and poorly understood mechanisms of invasion, growth and reproduction (Han and Weiss, 2017). *Blastocystis* spp. are ubiquitous anaerobic eukaryotes that have a unique phylogenetic origin but are widely present in various mammals, including humans (Scanlan and Stensvold, 2013). All these organisms have environmental stages required for the initiation of infection in new hosts, and were previously considered protozoa. Further controversies remain on the species structure, zoonotic potential, transmission routes, and pathogenicity/clinical significance of each group of organisms (Didier, 2005; Morris and Norris, 2012; Scanlan and Stensvold, 2013). Recently, with the development of various molecular tools for the identification and characterization and the availability of whole genome sequences, metagenomics tools, animal models and/or cultivation, there have been significant advances in our understanding of the biology and epidemiology of these controversial pathogens. The 2019–2020 Research Topic on Recent Advances in the Controversial Human Pathogens *Pneumocystis*, Microsporidia and *Blastocystis* has published 11 manuscripts, nine in Frontiers in Microbiology and two in Frontiers in Public Health. This topic aimed to gather articles from research groups who are active on these neglected and opportunistic pathogens. They highlight the potential of new tools and approaches in resolving some longstanding issues. These research articles are contributions from several major research groups in these three areas, significantly improving our knowledge of *P. jirovecii*, microsporidia and *Blastocystis* infections in different contexts.

## Pneumocystis

The articles gathered in this Research Topic addressed issues in epidemiology and risk factors of PCP in industrialized nations and low-income countries, especially on the genetic diversity of the pathogen. Attention was given to the use of modern diagnostic approaches. The pathogenesis of PCP and host defenses against *P. jirovecii* and its relationship with new therapies were discussed.

Knowing the prevalence and risk factors of PCP is a basic requirement for the prevention and control of the disease. Pereira-Díaz et al. conducted a nationwide study in Spain with this objective. It was a descriptive study of PCP patients registered in the Hospital Discharge Records Database of the country between 2008 and 2012. There was an increase in the annual incidence of PCP cases in non-HIV-infected patients and a decrease in HIV-infected patients. Risk factors identified in the HIV-negative group included hematological neoplasm, chronic lung diseases and non-hematological cancers. The mean mortality and the hospitalization costs observed were higher in non-HIV-infected patients with PCP than in HIV-infected patients with PCP. These observations indicate that PCP is an emerging problem in non-HIV-infected patients.

In another retrospective study of specimens from PCP patients from Réunion (a French island in the Indian Ocean), French Guiana (a French South American territory) and Brest (Brittany, metropolitan France), Le Gal et al. compared multilocus genotypes of *P. jirovecii* among these very different populations. A multilocus sequence typing (MLST) based on three distinct loci of *P. jirovecii* was used for the analysis of specimens. The data generated revealed significant genetic diversity of *P. jirovecii* in the islands and the existence of geographically segregated pathogen populations.

Morilla et al. addressed the issue of colonization by *P. jirovecii*. They analyzed epidemiological data from PCP patients and *P. jirovecii*-colonized individuals in Spain and data on the distribution of superoxide dismutase (SOD) genotypes of *P. jirovecii* isolates. Only two *SOD* genotypes were found in the country, with one being the dominant genotype in the colonized individuals. *SOD* is a single copy nuclear gene commonly used in genotyping studies. Based on the data obtained, the authors concluded that although SOD-PCR is not an attractive tool for genotyping *P. jirovecii*, it could be useful in distinguishing colonization from PCP.

The diagnosis of PCP relies heavily on microscopic visualization of organisms or DNA detection in respiratory specimens obtained by invasive/costly techniques, which are difficult to implement in low-income countries. As an alternative, blood biomarkers, reflecting the host-pathogen interaction, were tested (Matos and Esteves, 2016). Elevated serum levels of (1-3)- β-D-Glucan (cell wall component of *P. jirovecii* cystic forms or ascus) were related to PCP and proposed as marker of the disease, especially in non-HIV-infected immunocompromised patients (Esteves et al., 2014; Matos and Esteves, 2016). Recent studies propose the immunodiagnosis of PCP, based on the detection of IgM anti-*P. jirovecii* antibodies, using recombinant synthetic antigens, as a new diagnostic alternative (Tomás et al., 2016, 2020). However, the relationship between serology and the detection of *P. jirovecii* DNA in respiratory specimens is not yet clear. In this topic, Tomás et al. presented an innovative approach based on the development of a prototype for point-of-care serological diagnosis of PCP. Synthetic multiepitope antigens were designed, produced, and conjugated to gold nanoparticles. They were used in the development of two strip-based bionanodiagnostic assays for the specific detection of IgM against *P. jirovecii* in patient sera. This technology may offer an alternative to the conventional diagnosis of PCP, reducing the use of invasive procedures in collecting respiratory specimens. This new PCP diagnostic tool could be particularly useful in low-income countries, where this disease is emerging.

PCP therapy remains a challenge. The contribution of innate immunity to *Pneumocystis*-induced pathology is largely unexplored. Excess mucus and goblet-cell-derived CLCA1 protein activation appear to be associated with *Pneumocystis* infection in immunocompetent infants and animal models, and innate immunity related airway mucus responses may play a relevant role in the immunopathology of PCP. Existing treatment of PCP depend on anti-*Pneumocystis* drugs plus steroids that reduce cellular responses. Using a steroid-induced-immunosuppression rat model of PCP, Pérez et al. documented *Pneumocystis*-related pulmonary edema and progressive goblet-cell-derived CLCA1-related immunopathology. After the administration of the potent CLCA1-blocker Niflumic acid there were progressive reversal of mucous changes and improved animal survival. CLCA1 blockers could be used as an adjunctive therapy to improve clinical outcome, especially in steroid-induced PCP.

## Microsporidia

Microsporidia have an environmental stage, the spore, which contains a coiled polar tube anchored to the spore wall. It is the developmental stage used in transmission of microsporidia and invasion of host cells. Once the spores are ingested, the polar tube inside ejects, delivering the sporoplasm into host cells (Tamim El Jarkass and Reinke, 2020). The invasion mechanism of microsporidia remains poorly explored. In the Research Topic, Han B. et al. reviewed recent developments in the structure and composition of the spore wall and polar tube and the invasion process of microsporidia. The involvement of various polar tube proteins in the invasion of host cells was discussed in detail.

Immune responses to microsporidia have attracted recent attention. Studies in this area were done mostly with the three human-pathogenic *Encephalitozoon* species, which have various rodent models and can be cultured easily. Another review by Han Y. et al. has summarized recent understandings of the innate and acquired immune responses to microsporidia, especially the role of various immune cells (macrophages, dendritic cells, natural killer cells, etc.), the toll-like receptor-MyD88 pathway in innate immunity, and antibody, CD4+ lymphocyte and CD8+ lymphocyte responses in acquired immunity against microsporidia.

*Enterocytozoon bieneusi* is another major human-pathogenic microsporidian species, responsible for over 90% human microsporidiosis cases (Li and Xiao, 2021). Sequence analysis of the ribosomal internal transcribed spacer (ITS) has identified over 500 genotypes of various host ranges and zoonotic potential. They belong to at least 11 genogroups, with Group 1 containing most genotypes from humans and many genotypes from animals. Other groups have more restricted host ranges, therefore, representing host-adapted *E. bieneusi* populations. Therefore, genetic characterizations of isolates have been used in studies of the transmission dynamics, infection sources, and public health significance of *E. bieneusi* in humans and animals (Li et al., 2019). As a result, molecular epidemiology is a very active area in microsporidiosis research (Matos et al., 2012).

In the collection of articles for the Research Topic, three of the five ones on microsporidia were on molecular characterizations of *E. bieneusi*, all from the same research group. Data from the three studies suggest that domestic animals are infected with diverse *E. bieneusi* genotypes. In a study conducted on seven large-scale pig farms in Xinjiang, China by Li et al., a very high prevalence (48.6% or 389/801) of *E. bieneusi* was found. Altogether, 15 Group 1 genotypes were found, with the pig-adapted genotypes EbpA (*n* = 129) and EbpC (*n* = 168) as the dominant ones. These two genotypes and six other Group 1 genotypes were also found in a study by Zhao et al. of *E. bieneusi* in racehorses. A low frequency (prevalence = 4.8% or 30/621) of *E. bieneusi* was found in animals in 17 equestrian clubs in 15 Chinese cities. Among the *E. bieneusi* genotypes found, horse 1 (*n* = 16) was the dominant genotype. In addition to the Group 1 genotypes, one genotype each from Group 2 and Group 6 were found in 1–2 animals. In contrast, in another report by Wang et al., 11 *E. bieneusi* genotypes were found in dairy cattle (prevalence = 14.2% or 501/3527) in three provinces in China, with two bovine-adapted Group 2 genotypes I (*n* = 226) and J (*n* = 225) as the dominant genotypes. Therefore, cattle, pigs and horses in China are mostly infected with different groups of ITS genotypes of *E. bieneusi*. This has important implications in understanding cross-species transmission of *E. bieneusi*.

## Blastocystis

The pathogenicity of *Blastocystis* spp. remains controversial. Some major contributors to this debate include the influences of co-infections, microbiome, and subtype identity of *Blastocystis* spp. (Deng et al., 2021). In one study presented in the Research Topic by Betts et al., the subtype identity of *Blastocystis* spp., concurrence of *Cryptosporidium* spp., *Eimeria* spp., *Isospora* spp., *Entamoeba* spp., and *Giardia* spp. were examined in 231 fecal samples collected from asymptomatic animals in two conservation parks in the United Kingdom. Among the 38 vertebrate species examined, 47.4% (18/38) were positive for *Blastocystis* spp. Altogether, 10 known subtypes were identified, with ST2 as the most common one (31.4% or 80/255). Among the animal species with significant *Blastocystis*-positive samples, voles were mainly infected with ST4 (89.7% or 78/87), while non-human primates were mostly infected with ST1 (24.8% or 27/109), ST2 (56.9% or 62/109), and ST3 (11.0% or 12/109). Of the 81 *Blastocystis*-positive samples analyzed for other protists, 43 (53.1%) were positive for co-pathogens. These data suggest that co-infection and subtype identity should be considered in studies of pathogenicity of *Blastocystis* spp. in humans and animals.

## Author Contributions

Both authors listed have made a substantial, direct and intellectual contribution to the work, and approved it for publication.

## Conflict of Interest

The authors declare that the research was conducted in the absence of any commercial or financial relationships that could be construed as a potential conflict of interest.

## Publisher's Note

All claims expressed in this article are solely those of the authors and do not necessarily represent those of their affiliated organizations, or those of the publisher, the editors and the reviewers. Any product that may be evaluated in this article, or claim that may be made by its manufacturer, is not guaranteed or endorsed by the publisher.
